# Exploiting Clinical Trial Data Drastically Narrows the Window of Possible Solutions to the Problem of Clinical Adaptation of a Multiscale Cancer Model

**DOI:** 10.1371/journal.pone.0017594

**Published:** 2011-03-03

**Authors:** Georgios S. Stamatakos, Eleni C. Georgiadi, Norbert Graf, Eleni A. Kolokotroni, Dimitra D. Dionysiou

**Affiliations:** 1 In Silico Oncology Group, Institute of Communication and Computer Systems, School of Electrical and Computer Engineering, National Technical University of Athens, Athens, Greece; 2 Universität des Saarlandes, Klinik für Päd, Onkologie und Hämatologie, Homburg, Germany; University of Pennsylvania, United States of America

## Abstract

The development of computational models for simulating tumor growth and response to treatment has gained significant momentum during the last few decades. At the dawn of the era of personalized medicine, providing insight into complex mechanisms involved in cancer and contributing to patient-specific therapy optimization constitute particularly inspiring pursuits. The *in silico* oncology community is facing the great challenge of effectively translating simulation models into clinical practice, which presupposes a thorough sensitivity analysis, adaptation and validation process based on real clinical data. In this paper, the behavior of a clinically-oriented, multiscale model of solid tumor response to chemotherapy is investigated, using the paradigm of nephroblastoma response to preoperative chemotherapy in the context of the SIOP/GPOH clinical trial. A sorting of the model's parameters according to the magnitude of their effect on the output has unveiled the relative importance of the corresponding biological mechanisms; major impact on the result of therapy is credited to the oxygenation and nutrient availability status of the tumor and the balance between the symmetric and asymmetric modes of stem cell division. The effect of a number of parameter combinations on the extent of chemotherapy-induced tumor shrinkage and on the tumor's growth rate are discussed. A real clinical case of nephroblastoma has served as a proof of principle study case, demonstrating the basics of an ongoing clinical adaptation and validation process. By using clinical data in conjunction with plausible values of model parameters, an excellent fit of the model to the available medical data of the selected nephroblastoma case has been achieved, in terms of both volume reduction and histological constitution of the tumor. In this context, the exploitation of multiscale clinical data drastically narrows the window of possible solutions to the clinical adaptation problem.

## Introduction

The last few decades have witnessed an increased interest of the scientific community into the development of computational models for simulating tumor growth and response to treatment [1]–[3]. At the beginning of the era of personalized medicine, sophisticated multiscale models yield valuable quantitative insights into complex mechanisms involved in cancer and may ultimately contribute to patient-specific therapy optimization.

The major modeling approaches can be distinguished into predominantly continuous and predominantly discrete models. Predominantly continuous models rely primarily on differential equations to describe processes such as diffusion of molecules, changes in tumor cell density and invasion of tumor cells into the surrounding tissue [4]–[9]. Predominantly discrete modeling considers several discrete states in which cells may be found and possible transitions between them, governed by “decision calculators”, such as cytokinetic diagrams and agent-based techniques [10]–[18]. Discrete models are usually represented by cellular automata of several forms and variable complexity (grids of cells or groups of cells, in which a finite number of states and a set of evolution and interaction rules are defined). Due to the hypercomplexity of cancer-related topics, each modeling approach is intrinsically able to satisfactorily address only some of the aspects of this multifaceted problem. Ultimate goal of clinically-oriented cancer simulation models is their eventual translation into clinical practice, which entails a) thorough sensitivity analyses, in order to both comprehend and validate their behavior, and at the same time gain further insight into the simulated mechanisms, in a more quantitative way, and b) an adaptation and validation process based on real clinical data.

This paper investigates the behavior of an actual clinical trial-driven model simulating the response of nephroblastoma tumors to preoperative chemotherapy. Nephroblastoma (also termed Wilms' tumor) is the most common renal malignancy in children [19], [20]. Indicative results of an in-depth sensitivity analysis of the model regarding the effect of critical mechanisms involved in the dynamics of the biological system are presented, along with a proof of principle, successful adaptation study to an actual clinical Wilms' tumor case, drawn from the SIOP 2001/GPOH trial [21], [22]. The model is in the process of clinical adaptation and validation within the framework of the EC-funded project “ACGT: Advancing Clinicogenomic Trials on Cancer (FP6-2005-IST-026996)”.

## Methods

### Ethics statement

This research is approved by the Ethical Committee of the Aerztekammer des Saarlandes (104/10 from 20 July 2010). Written informed consent was given by the parents of the child whose clinical data were used in this work.

### General features of the simulation model

The model is a predominantly discrete, clinically-oriented multiscale cancer model of solid tumor response to chemotherapy [23], [24], stemming from previous work of the *In Silico* Oncology Group (ISOG), National Technical University of Athens (NTUA). A “top-down” simulation approach is formulated [25], [26]; the method starts from the macroscopic imaging data (a high biocomplexity level) and proceeds towards lower biocomplexity levels. When there is a need for an upwards movement in the biocomplexity scales, a summary of the available information pertaining to the previous lower level is used. The clinical orientation of the model has been a fundamental guiding principle throughout its development. Available medical data can be exploited, in order to strengthen patient-individualized modeling. The model is under continuous refinement in the framework of clinical trials.

### Basic algorithmic notions

**Figure 1:**
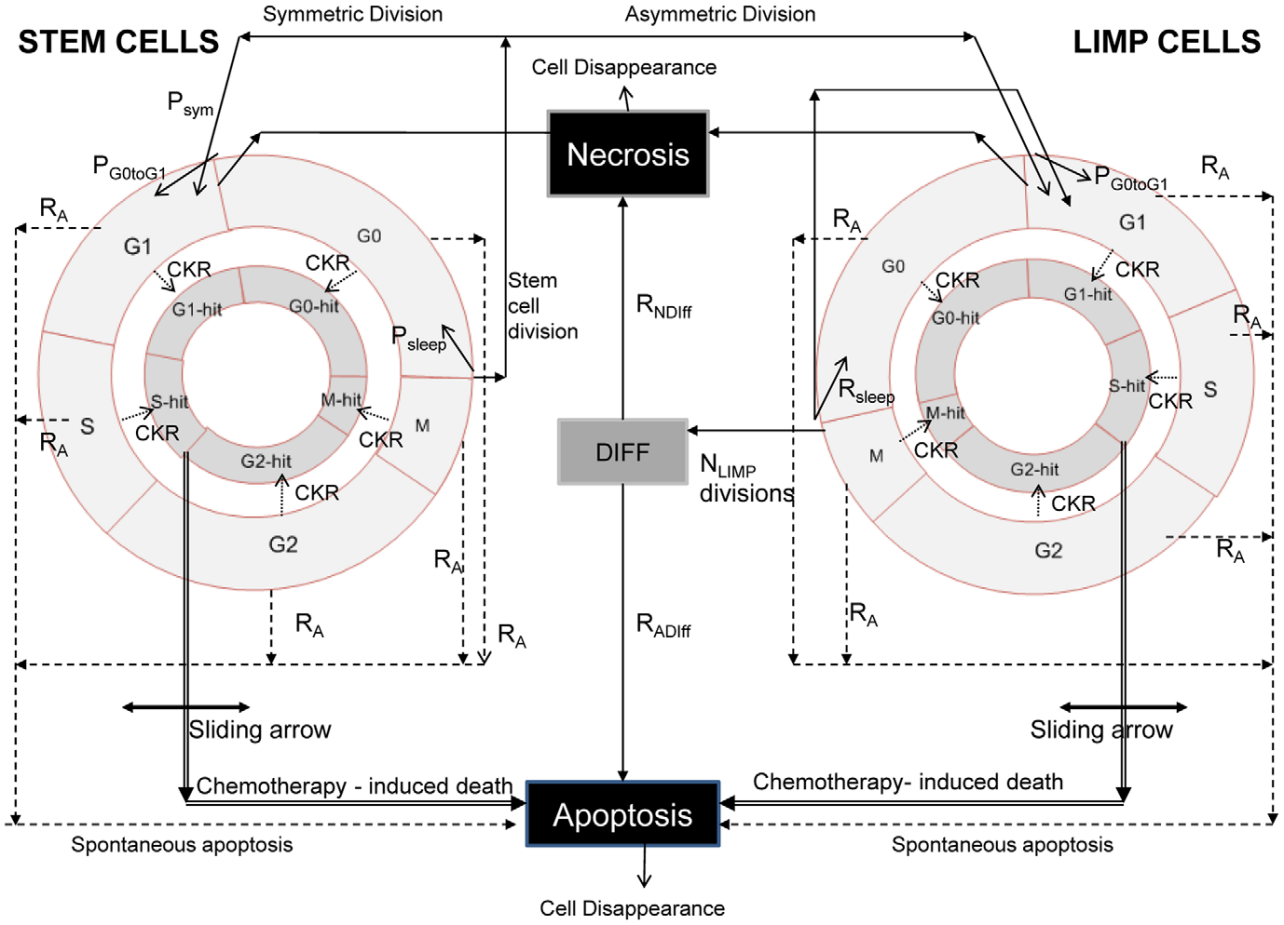
Generic cytokinetic model for tumor response to chemotherapy. The generic cytokinetic model used. LIMP: LImited Mitotic Potential cells. DIFF: terminally differentiated cells. G1: Gap 1 phase. S: DNA synthesis phase. G2: Gap 2 phase. M: Mitosis. G0: dormant phase. Hit: cells lethally hit by chemotherapy. The arrow indicating chemotherapy-induced death is a sliding arrow, with position dependent on drug pharmacodynamics. For a definition of the depicted model parameters see Table 1.

The following five categories (or “equivalence classes”) of cancer cells are considered in the model: stem cells (cells of unlimited mitotic potential), LIMP cells (LImited Mitotic Potential or committed progenitor cells, which can perform a limited number of mitoses before terminal differentiation), terminally differentiated cells, apoptotic and necrotic cells. The various cell cycle phases (G1, S, G2, M) and the dormant (G0) phase constitute subclasses in which stem or LIMP cells may reside. Figure 1 depicts the developed cytokinetic model, which incorporates several biological phenomena that take place at the cellular level:

- Cycling of proliferating cells through the successive cell cycle phases.
- Symmetric and asymmetric modes of stem cell division.
- Terminal differentiation of committed progenitor cells after a number of mitotic divisions.
- Transition of proliferating cells to the dormant phase due to inadequate supply of oxygen and nutrients.
- Reentering of dormant cells into the active cell cycle due to local restoration of oxygen and nutrient supplies.
- Cell death through spontaneous apoptosis.
- Cell death through necrosis (due to prolonged oxygen and nutrients' shortage).
- Cell death due to chemotherapy-induced apoptosis.

Table 1 presents the corresponding tumor dynamics model parameters.

**Table 1:** Tumor dynamics model parameters studied in the sensitivity analyses.

Symbol (units)	Definition	Reference Value	References	T1	T2	T3	T4
Tc (h)	Cell cycle duration	23.0	[39]	23.0	40	23.0	55
T_G0_ (h)	G0 (dormant phase) duration, i.e. time interval before a dormant cell dies through necrosis	96	[40]	96	96	96	40
T_N_ (h)	Time needed for necrosis to be completed and its lysis products to be eliminated from the tumor	20	[10], [15], [41]	20	20	20	120
T_A_ (h)	Time needed for apoptosis to be completed and its products to be eliminated from the tumor	6	[42], [43]	6	6	6	6
R_A_ (h^−1^)	Apoptosis rate of living stem and LIMP tumor cells (fraction of non-differentiated cells dying through apoptosis per hour)	0.001	Derived from T_A_, based on [42], [43]	0.001	0.0008	0.001	0.001
R_ADiff_ (h^−1^)	Apoptosis rate of differentiated tumor cells per hour	0.003		0.003	0.003	0.003	0.05
R_NDiff_ (h^−1^)	Necrosis rate of differentiated tumor cells per hour	0.001	Derived from T_N_, based on [10], [41]	0.001	0.001	0.001	0.05
P_G0toG1_	The fraction of stem or LIMP cells having just left the G0 compartment that re-enter the cell cycle	0.01		0.01	0.01	0.01	0.01
N_LIMP_	The maximum number of mitoses that a LIMP cell can perform before becoming terminally differentiated	3		3	3	3	3
P_sym_	Fraction of stem cells that perform symmetric division.	0.45		0.71	0.45	0.45	0.76
P_sleep_	Fraction of cells that enter G0 phase following mitosis	0.28		0.40	0.28	0.28	0.36
CKR_VCR_	Cell kill ratio for the specific vincristine dose	0.3	Derived based on [35], [36]	0.3	0.3	0.36	0.33
CKR_ACT_	Cell kill ratio for the specific actinomycin-D dose	0.2	Derived based on [37], [38]	0.2	0.2	0.34	0.22
CKR_TOTAL_*	Combined cell kill ratio of the two drugs (dependent parameter)	0.5	Additive drug effect considered	0.5	0.5	0.7	0.55

Definition of tumor dynamics model parameters, reference values and corresponding literature references, and values assigned for the implementation of four virtual tumors. T1: Tumour T1, T2: Tumour T2, T3: Tumour T3, T4: Tumour T4. CKR_total_ is not an independent parameter of the model.

In order to simulate chemotherapy-induced cell death, lethally hit cells are assumed to enter a rudimentary cell cycle leading to apoptotic death. Cell cycle-specific, cell cycle-non specific, cell cycle phase-specific and cell cycle phase-non specific drugs can be simulated, as detailed in [23]. “Marking” of a cell as hit by the drug is assumed to take place at the instant of drug administration. However, its actual time of death is dictated by the specific drug's pharmacokinetics and pharmacodynamics. The numbers of cells hit by the drug are computed through the utilization of the cell kill ratio (CKR) parameter (CKR = 1-cell survival fraction), defined as the percentage of lethally hit cells after each drug administration. A diversification of chemotherapeutic resistance between tumor stem and non-stem cells can be easily achieved through the use of different values of the corresponding CKR parameters.

For a relatively short time interval compared to the tumor's lifetime (such as the duration of a simulated chemotherapeutic schedule) the various cell category/phase transition rates are considered approximately constant and reflect the means of the actual cell category/phase transition rates over the interval.

### Virtual tumor spatiotemporal initialization

A three-dimensional cubic mesh discretizing the region of interest is considered. The elementary volume of the mesh is called geometrical cell (GC). Each GC of the tumor accommodates initially a number of biological cells (NBC), which is defined based on typical solid tumor cell densities (e.g. 10^9^ cells/cm^3^) [27], unless more specific information for a particular tumor is available. The cells initially residing within each GC of the mesh are distributed into the five classes and subclasses mentioned above. The technique used for the tumor's constitution initialization is critical, in order to avoid latent artificial tumor growth behaviors, as previously described in [23], [24].

The model supports the division of tumor area into different metabolic regions (e.g. necrotic and proliferative) based on pertinent imaging data and the handling of each region separately. In this case different values of specific model parameters can be assigned to each region.

### Virtual tumor spatiotemporal evolution

At each time step the discretizing mesh is scanned and the basic cytokinetic, metabolic, pharmacokinetic/pharmacodynamic and mechanical rules that govern the spatiotemporal evolution of the tumor are applied. Practically, each complete scan can be viewed as consisting of two mesh scans, as described in [23]. Briefly speaking, the first scan aims at updating the state of each GC, by applying the rules of the cytokinetic model of Figure 1. The second scan serves to simulate tumor expansion or shrinkage, based on the principle that, throughout a simulation, the total population of a GC is allowed to fluctuate between a minimum and a maximum value, defined in relation to the initial typical GC cell content. At each time step, checks of each GC total population designate whether the total cell number is above/below the predefined max/min thresholds and, if necessary, specially-designed cell content shifting algorithms “create” or “delete” GCs and thereby lead to tumor expansion or shrinkage, respectively.

A simplified flowchart of the entire simulation procedure is provided as supporting material (Figure S1). A detailed description of technical issues involved in the construction of an integrated simulation platform incorporating image processing, visualization and grid execution facilities will be the topic of a separate paper. Initial presentations can be found in [26], [28], [29].

### Nephroblastoma preoperative chemotherapy in the context of the SIOP/GPOH clinical trial

A thorough study of nephroblastoma literature preceded the simulations, so as to define -in conjunction with accumulated basic science and clinical experience-plausible reference values and value ranges of the various model parameters (Table 1).

**Figure 2:**
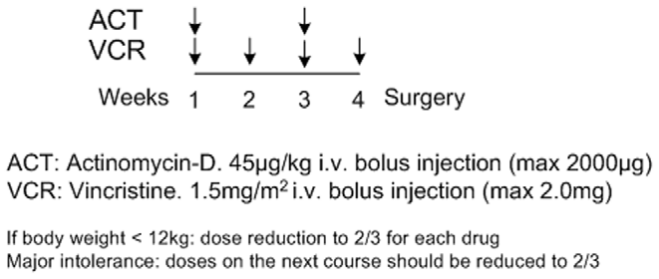
Chemotherapy treatment protocol. The simulated Wilms tumor preoperative chemotherapy treatment protocol of the SIOP/GPOH clinical trial.

A protocol of preoperative chemotherapy with a combination of actinomycin-D and vincristine for unilateral stage I-III nephroblastoma tumors, treated according to the SIOP 2001/GPOH clinical trial (Figure 2), in the framework of the ACGT project, has been specifically simulated in the present paper.

Vincristine's antineoplastic effect is basically attributed to its ability to bind to the protein tubulin, thereby destroying the functionality of the cell's microtubules, which form the mitotic spindle, and ultimately resulting in apoptotic cell death at mitosis (an M-phase specific drug) [30]–[32]. Therefore, in the simulation model vincristine is assumed to bind at cells at all cycling phases and lead to apoptosis at the end of M phase. Vincristine's toxicity is known to decrease with increasing tumor cell density (“inoculum effect”) [33].

Actinomycin-D is a cell cycle-nonspecific antitumor antibiotic that binds to double-stranded DNA through intercalation between adjacent guanine-cytosine base pairs [34]. It also acts to form toxic oxygen-free radicals, which create DNA strand breaks, inhibiting DNA synthesis and function. Based on the above, in the model actinomycin-D is considered to bind to cells at all phases (including G0) and lead to apoptosis at the end of the S phase.

The method used for the initial estimation of typical values of the cell kill ratios of vincristine and actinomysin-D is based on relevant pharmacokinetics and pharmacodynamics literature [35]–[38] (see Text S1).

According to the SIOP 2001/GPOH clinical trial protocol, vincristine i.v. bolus injection is directly followed by an i.v. bolus injection of actinomycin-D, with no delay in-between. As a first approximation, an additive drug effect of vincristine and actinomycin-D has been assumed for all active cell cycle phases. For dormant cells only actinomycin-D exerts a cytotoxic effect.

## Results

### Cellular level-mechanisms with major impact on nephroblastoma response to chemotherapy

The results of the sensitivity analyses performed permitted the sorting of the model's parameters –and hence of the corresponding biological mechanisms- according to the magnitude of their effect on selected outputs. These are cellular-level biological mechanisms, but are governed by—and thus summarize—various genetic determinants which may diversify the tumor phenotype, prognosis and response to therapy for each particular clinical case. More specifically, all model parameters pertaining to tumor dynamics have been studied (twelve parameters in total, see Table 1). The remaining few model parameters (see Table S1) are miscellaneous parameters unrelated to the tumor's dynamics. The simulation outcome considered was the tumor volume reduction after chemotherapy treatment, since this is a typical measure of the response to preoperative chemotherapy treatment in the clinical setting [19], [20]. The details of the sensitivity analysis approach adopted are presented as supporting material (Text S2).

**Figure 3:**
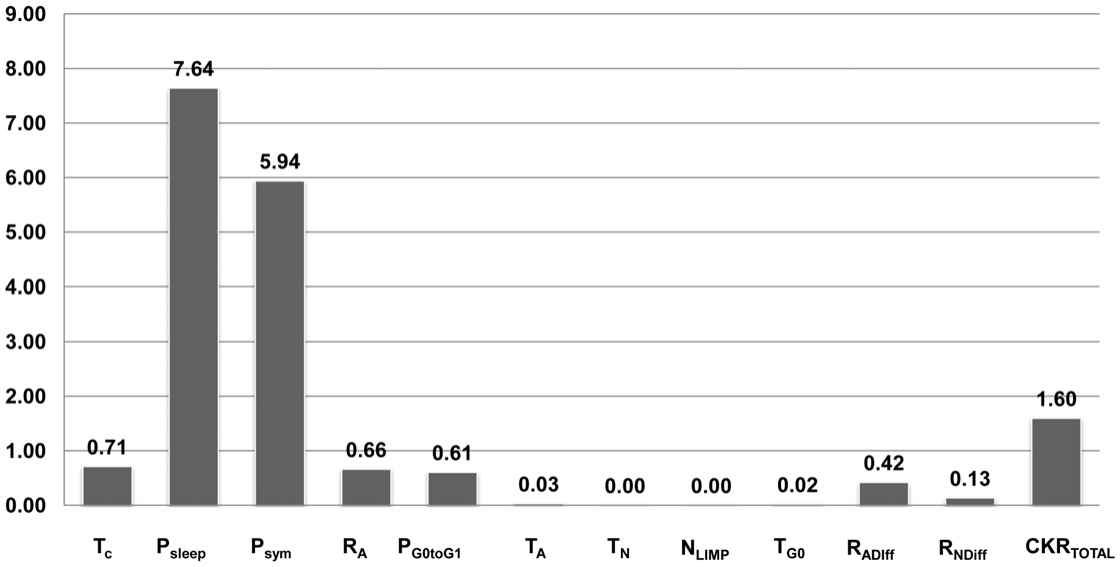
Sorting of the model parameters according to their effect on chemotherapy-induced tumor shrinkage. Sorting of the model parameters according to their effect on chemotherapy-induced tumor shrinkage. For a definition of the depicted model parameters see Table 1. SC: Sorting Criterion (see Text S2).

As shown in Figure 3, the two biological mechanisms mostly implicated in the result of therapy are:

- The oxygen and nutrients availability status of the tumor (as expressed mainly by the fraction of cells entering the dormant phase following mitosis - P_sleep_), and
- The balance between the symmetric and asymmetric modes of stem cell division, reflecting intrinsic properties of stem cells and/or extrinsic controls from their microenvironment (represented by the fraction of stem cells that divide symmetrically - P_sym_)

Other parameters completing the picture of tumor response to therapy, but with significantly reduced impact on the selected outcome compared to the previous two, are:

- The cytotoxicity of the chemotherapeutic agents (reflected by their total cell kill ratio – CKR_total_)
- The cell cycle duration - T_c_
- The apoptosis rate of living stem and committed progenitor (LIMP) tumor cells - R_A_.
- The fraction of the dormant cells having just left the G0 compartment that re-enter the cell cycle -P_G0toG1_ (which constitutes a further way through which the oxygenation and nutrients' availability status of the tumor plays a role in the model).

**Figure 4:**
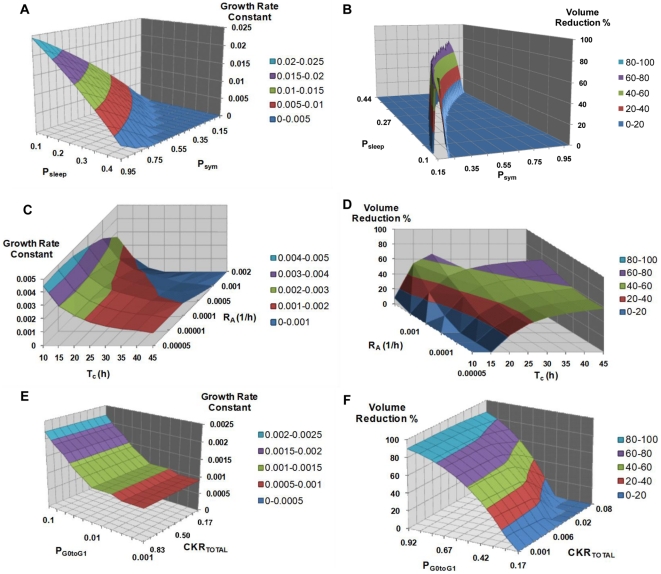
Selected combined effects of several model parameter combinations. Combined effects of selected parameter combinations on tumor free growth rate (first column) and volume reduction after therapy (second column). Different colors correspond to distinct *ranges* of the growth rate constant value or the tumor volume reduction percentage. **Panels A, B**: Combined effect of Psym and Psleep. **Panels C,D**: Combined effect of Tc and R_A_. **Panels E,F**: Combined effect of CKR_total_ and P_G0toG1_. For a definition of the depicted model parameters see Table 1.

An additional parametric analysis is presented in Figure 4, involving the previously defined six most critical parameters which largely complete the picture of the tumor's response to treatment in terms of volume reduction (i.e. P_sleep_, P_sym_, CKR_total_, T_c_, R_A_, P_G0toG1_). The combined effects of a number of parameter dyads on the reduction percentage of a chemotherapeutically treated tumor and on the growth rate constant characterizing its free growth or re-growth after completion of therapy have been studied. The considered parameter dyads are: i) P_sym_ and P_sleep_, ii) T_C_ and R_A_, and iii) CKR_total_ and P_G0toG1_.

For tumor regrowth after therapy studies, an exponential free growth pattern has been considered, which in fact approximates a segment of the Gompertzian curve, as explained in [23]. The areas that appear in the graphs of Figure 4 show only combinations of biologically relevant parameter values leading to tumors that exhibit monotonic behavior for the case of free growth [23], [24] and tumors displaying volume reduction after therapy for the case of treatment.

Figure 4A shows the combined effect of P_sym_ and P_sleep_ on the growth rate of the tumor. An intuitive observation is that a tumor is more aggressive (with a higher growth rate constant) for higher values of P_sym_ and lower values of P_sleep_, which points out the counteracting effect of the two mechanisms. The growth rate “isosurfaces” (here defined as distinct *ranges* of the growth rate constant values and indicated by distinct colors) form parallel stripes, implying that the effect of the combination of the two parameters retains the same character over the entire value space considered.

Figure 4C shows the combined influence of T_C_ and R_A_. Virtual tumors with prolonged cell cycle duration are less aggressive (with a lower growth rate constant) than tumors with short cell cycle durations. This difference becomes greater for higher values of the spontaneous apoptosis rate. The tumor growth rate “isosurfaces” appear almost parallel to the axis of R_A_ for low values of T_C_: the influence of spontaneous apoptosis on the growth rate of the tumor is much less pronounced than the effect of the cell cycle duration (which is in accordance with the results presented in Figure 3).

In Figure 4E a biologically anticipated finding is that tumors with higher P_G0toG1_ values have higher growth rate constants. Also, as expected, the drugs' cell kill ratio has no effect on the tumor free growth rate; therefore, “isosurfaces” parallel to the axis of the CKR parameter appear in this case.

In Figure 4B an isoline of maximum volume reduction is discernible. A sharp decrease in the output is observed when changing the parameter values from those that lead to that maximum reduction, which is characteristic of the pronounced sensitivity of the output on the values of these two parameters, in accordance with the results of Figure 3. Parallel “isosurfaces” are another characteristic of the output in this case too.

Figure 4D indicates larger volume reductions for tumors with high values of T_C_ and high values of R_A_. Finally, as shown in Figure 4F, an increased CKR of the combination of the chemotherapeutic agents (i.e. increased cytotoxicity) leads intuitively to greater tumor volume reductions. The volume reductions are slightly higher for higher values of P_G0toG1_.

### Clinical adaptation of the model: a proof of principle simulated clinical case

A clinical case of nephroblastoma from the SIOP 2001/GPOH trial has been selected and the corresponding anonymized imaging and clinical data have been collected. The outer boundary of the tumor based on two sets of MRI images has been provided for two time instants, the first one corresponding to the time of diagnosis (4 days before the beginning of the chemotherapy treatment) and the second one 3 days after the last drug administration. At this first clinical adaptation step, the spatial distribution of macroscopically distinct tumor subregions was not available for the particular clinical case and therefore an equivalent tumor of the same constitution in terms of cell categories population numbers has been considered. Based on the imaging data, chemotherapy has achieved tumor shrinkage equal to 73%. Post-surgery histological data indicated a highly malignant, blastemal type of tumor, with a regression/necrosis component after chemotherapy approximately equal to 60% and a 100% blastemal component for the remaining viable tumor. The available histological information for the particular tumor has been used in the model so as to provide a means of appropriately adjusting the corresponding populations percentages in the equivalent homogeneous virtual tumor considered.

Results of sensitivity analyses such as those presented in the previous section, have provided guidance for the selection of the model parameter values so as to succeed in implementing a virtual tumor consistent with the actual clinical data, both in terms of tumor volume measurements and histological constitution of the tumor. Four virtual tumor scenarios in agreement with the tumor volume imaging data measurements have been specified. The values assigned to the model parameters for the implementation of the four virtual tumor scenarios are presented in Table 1. Derived tumor characteristics (doubling time, growth fraction etc.) and resultant therapy-induced shrinkages are presented in Tables 2 and 3. Taking into account all the uncertainties in the medical and literature data that have been used, Table 2 should be interpreted as indicating approximate values of the various tumor properties.

**Table 2:** Initial virtual tumors' characteristics.

Resultant initial tumor characteristics	Typical tumor*	T1	T2	T3	T4
Growth Rate Constant, k (h^−1^)	0.001	0.0004	0.0004	0.001	0.0014
Volume Doubling Time, T_d_ = ln2/k (days)	29	72	72	29	21
Initial percentage of proliferating cells (Growth Fraction) (%)	14	15	19	14	37
Initial percentage of dormant cells (%)	18	36	16	18	14
Initial percentage of stem cells (%)	12	32	14	12	35
Initial percentage of LIMP cells (%)	20	19	21	20	16
Initial percentage of differentiated cells (%)	62	40	59	62	2
Initial percentage of dead cells (%)	6	9	6	6	47

Initial tumor characteristics and volume reduction percentages for the four virtual tumor scenarios, defined by the parameter values given in Table 1. The total tumor cell population is derived by adding the subpopulations of proliferating cells, dormant cells, differentiated cells and dead cells or, alternatively, the subpopulations of stem cells, LIMP cells, differentiated cells and dead cells.

*The column “typical tumor” presents the characteristics of a tumor implemented by assigning to all model parameters their assumed reference value; it does not constitute a solution for the simulation case considered.

**Table 3:** Final virtual tumors' characteristics and tumor volume reduction percentages.

Final tumor characteristics	Typical tumor*	T1	T2	T3	T4
	1 day after completion of therapy	3 days after completion of therapy			
Tumor volume reduction percentage (%)	56	72	72	72	73
Final percentage of proliferating cells (Growth Fraction) (%)	7	10	11	6	30
Final percentage of dormant cells (%)	13	27	11	11	12
Final percentage of stem cells (%)	7	23	8	7	29
Final percentage of LIMP cells (%)	13	14	14	10	13
Final percentage of differentiated cells (%)	74	55	73	78	1
Final percentage of dead cells (%)	6	8	5	5	57

Initial tumor characteristics and volume reduction percentages for the four virtual tumor scenarios, defined by the parameter values given in Table 1. The total tumor cell population is derived by adding the subpopulations of proliferating cells, dormant cells, differentiated cells and dead cells or, alternatively, the subpopulations of stem cells, LIMP cells, differentiated cells and dead cells.

*The column “typical tumor” presents the characteristics of a tumor implemented by assigning to all model parameters their assumed reference value; it does not constitute a solution for the simulation case considered.

The volume reduction for these simulated tumors is equal to 72% for T1, T2, and T3, and 73% for T4. These results are in very good agreement with the imaging data-specified volume shrinkage of 73%. After having initially assigned reference values to all model parameters, exploratory perturbations have been performed in order to achieve agreement with clinical data. Tumor T1 has been derived by appropriately perturbing P_sym_ and P_sleep_, and tumor T2 by adjusting T_C_ and R_A_. The third scenario (T3) has been specified, by considering an initial tumor with all parameters kept at their reference values, apart form the total cell kill ratio, which has been adequately perturbed in order to fit the tumor volume measurements. As will be subsequently described, the final virtual scenario (T4) incorporates all necessary parameter perturbations to achieve full compliance with all medical and literature data.

Since all four virtual tumors are in good agreement with the data in terms of tumor volume reduction, they would be thought of as fairly good solutions of the simulation problem if no further information was available. Nevertheless, as revealed by the detailed tumor characteristics of these solutions, the corresponding tumors' subpopulation constitution and growth rate characteristics could be highly variable. To the best of our knowledge, all attempted adaptations of simulation models to clinical data reported up to now in the literature involve agreement in terms of tumor volume or total cell population, with the exception of modeling efforts that include a distinction between proliferating and quiescent cells [14] or oxic and hypoxic subpopulations [15]. In sharp contrast, the presented model, offers the possibility of a full clinical adaptation of all available information: both imaging and histological data. Its structure permits formulation of quantitative hypotheses regarding as yet unavailable data (e.g. initial tumor subpopulations), but which, very importantly, fulfill the constraints of the clinical information in hand.

Tumor T4 is a scenario fully satisfying the available histological constraints of the clinical case considered, with a post-chemotherapy population of dead cells close to 60% (≈57%) and a quite negligible population of differentiated cells (≈1%) since a blastemal type of tumor is being studied. At the same time, this scenario is in a rather good agreement with nephroblastoma literature regarding those tumor characteristics for which no clinical input was available: volume doubling time of 21 days (a range of 11–40 days is reported in literature [44]–[48]), pre-chemotherapy and post-chemotherapy growth fractions approximately equal to 37% and 30%, respectively, (corresponding percentages reported in literature for nephroblastomas of blastemal type [49]: 31–80% and 11–40%, respectively). Notwithstanding a) that parameter values outside ranges specified in literature could certainly not be excluded, due to both inter-patient variability and methodological issues related to the procedures used for their estimation, and b) that the estimated quantitative features of the tumors are only of an approximate character, the above observations demonstrate the basic philosophy of a possible procedure towards the selection of prevailing virtual scenarios, based on the combined use of the available for each patient case clinical and literature data. As the available information regarding a particular tumor's characteristics increases, further narrowing of the window of possible solutions is to be expected. Very importantly, virtual tumor T4 satisfies concurrently a considerable number of constraints which drastically limit the value range of the critical model parameters implicated in tumor response to therapy (e.g. P_sym_, P_sleep_). Our sensitivity analyses indicate that, under all the concurrent constraints considered, large deviations from the specified values of these critical parameters, and hence radically different solution characteristics, would not be expected, if an “exhaustive” solution search to the particular adaptation problem was attempted. Rather, different solutions would result mainly from alternative values in parameters that remain largely unspecified based on the available data. Such an example is the N_LIMP_ parameter, which currently remains unspecified based on the available data; an indication regarding the relative percentages of stem and committed progenitor cells would restrict the range of permitted perturbations in its value.

**Figure 5:**
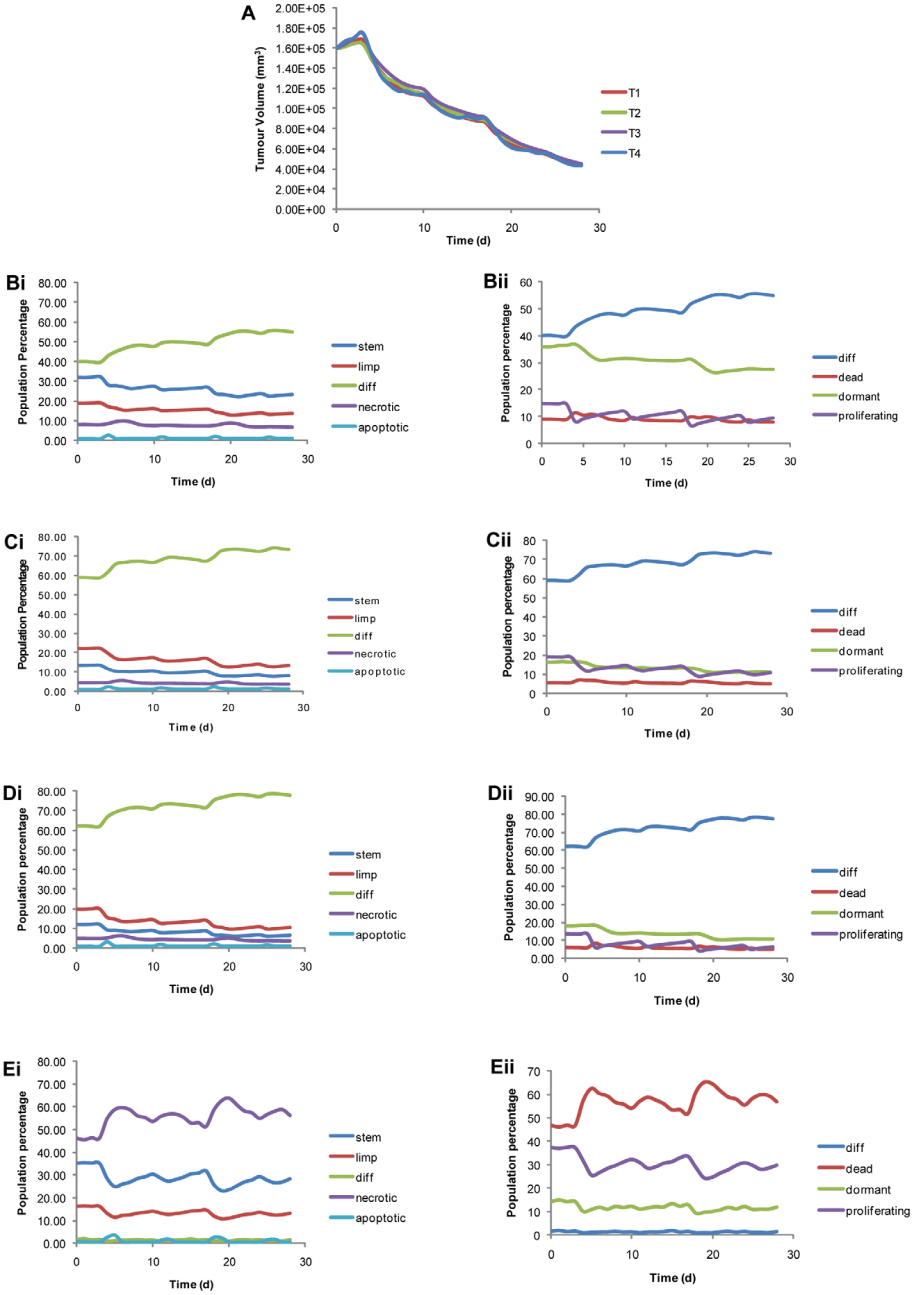
Time evolution of tumor volume and selected tumor subpopulations. **Panel A**: Time evolution of tumor volume for the four virtual scenarios of Table 1. **Panels Bi and Bii, Ci and Cii, Di and Dii, Ei and Eii**: Evolution over time of selected subpopulations of the tumors. The chemotherapeutic scheme of Figure 2 has been simulated. The drug administration instants are: day 3, day 10, day 17, day 24. Day 0: first MRI data set. Day 28: second MRI data set.

Bearing all this in mind, in Figure 5A the time course of the four virtual tumors is presented. As discussed, the final tumor volume is about the same for all tumors. Nevertheless, differences in the evolution over time are discernible among the studied cases (Figure 5B,C,D,E), since different tumor dynamics parameter values lead to different initial cell subpopulations and have implications for their evolution over time and the effect of therapy. Numerous interesting theoretical observations could be made based on Figure 5 (see also Figure S2); the following, though, stand out:

- Since P_sym_ and P_sleep_ are the two parameters with the major impact on the tumor's evolution, the use of similar values for these parameters in different virtual tumors results in quite similar patterns of evolution over time for all cell subpopulations. This is particularly evident in the case of T2 and T3 tumors (which have exactly the same values of P_sym_ and P_sleep_).
- T1 and, particularly, T4 are characterized by the highest stem cell content, as they have the highest symmetric division fraction values. It is interesting that the currently derived high stem cell content of T4 correlates with the high malignancy and poor prognosis of nephroblastomas of blastemal type, particularly so in the context of recent reports in literature suggesting that individual tumors that are, at the histopathological level, relatively undifferentiated may contain higher proportions of stem cells than their more differentiated counterparts [50]. Furthermore, recent evidence suggests that within some tumors cancer stem cells may be as numerous as the non-stem cells with which they co-exist [50].
- T1's high P_sleep_ value and high T_G0_ value lead to a significantly higher initial percentage of dormant cells compared to the rest of the virtual tumors.
- T2 and T3 due their lower P_sym_, have higher fractions of differentiated cells compared to T1.
- The large duration of necrosis in the case of T4 is directly related to the large dead cell component of this tumor.

## Discussion

The central focus of this work has been a thorough sensitivity analysis of the simulation model, revealing the relative importance of its parameters. A sorting of the parameters, and hence of the corresponding cellular-level biological mechanisms, with major impact on the simulation outcome has been performed. Indicative parametric investigations that shed light on complex parameter interrelations, which often cannot be grasped intuitively, have been presented. The two biological mechanisms mostly implicated in the result of therapy are the oxygenation and nutrients availability status of the tumor and the balance between the symmetric and asymmetric modes of stem cell division. These results constitute part of an extensive series of such parametric studies, aiming at deepening and advancing quantification of our understanding of tumor response to chemotherapeutic treatment in the nephroblastoma and, more specifically, the SIOP/GPOH clinical trial context.

A clinical case of nephroblastoma from the SIOP 2001/GPOH trial has been selected and by using plausible values of the model parameters derived from clinical experience and relevant literature, an excellent fit of the model to the available clinical data has been achieved in terms of both volume reduction and histological constitution of the tumor. Furthermore, derived critical tumor characteristics for which no direct clinical information was available are in good agreement with relevant nephroblastoma literature. Whereas various attempts of model adaptations to volumetric data have already been reported in the literature, agreement with clinical data in tumor volumetric terms alone may mask tumors with radically different characteristics. The potential to readily exploit additional data available in the context of clinical trials, thereby narrowing the window of possible solutions, is a particularly distinctive feature of the ISOG model.

The fitting of the selected nephroblastoma case to the clinical data serves as a proof of principle example, demonstrating the basic philosophy of a possible procedure towards the selection of prevailing virtual scenarios, based on the combined use of the available multiscale clinical and literature data. As the available information regarding a particular tumor's characteristics increases, further narrowing of the window of possible solutions is to be expected. Availability of multiscale medical data imposes constraints on model parameter values. Conversely, after adequate “tuning” the simulation results could give valuable hints concerning tumor characteristics for which actual estimations might be missing in each case considered.

Major scientific challenge for the ISOG modeling efforts is the eventual translation of its detailed multiscale cancer models to clinical practice. The use of anonymized data before and after treatment constitutes the basis for the clinical adaptation and validation process. As more and more sets of medical data are exploited the reliability of the model results is expected to increase and patient-individualized modeling to be strengthened. In future versions of the model, the individual patient's serum response to specific tumor antigens will be considered as well: in the context of the ACGT project, possible correlations of the autoantigen pattern with tumor histology (i.e. blastemal, epithelial, and stromal cell fractions) are under investigation [26]. Future versions of the model will also handle cases of inhomogeneous tumors with macroscopically/metabolically distinct regions. The integrated simulation system, (incorporating image processing, visualization, grid execution and other technical facilities) has been termed “Oncosimulator” [25]. Two “oncosimulators” are currently being developed by ISOG, clinically adapted and validated using real clinical trial multiscale data within the framework of the EC funded projects ACGT [25] and “Contra Cancrum” (FP7-ICT-2007-2-223979) [51].

It is envisaged that, at a later stage, after the completion of the necessary adaptation and validation procedure, such simulation platforms could support the design of new experiments or clinical trials, by identifying important scientific questions and open issues, brought forward through an in-depth understanding of the system; they could even offer the potential for studying various biological mechanisms and interactions without performing time-consuming and costly laboratory experiments or clinical trials. Designing some of these clinical trials or experiments is extremely difficult, if at all feasible, as they would have to refer to the basic science level.

*In silico* oncology holds much promise in the field of cancer research. It certainly has not yet reached its full potential, multiple challenges of diverse nature exist and many unresolved issues remain to be addressed. Nevertheless, the presented successful initial adaptation step lends support that the ISOG modelling efforts are indeed on a viable track towards clinical adaptation.

## Supporting Information

Figure S1 **Simplified flowchart of the simulation procedure.** Simplified flowchart of the simulation procedure. GC: Geometrical Cell. Y:Yes, N:No.
(TIF)

Figure S2 **Time evolution of various tumor subpopulations.** Alternative presentation of various tumor subpopulations for the four virtual tumor scenarios implemented (T1: Tumor1, T2: Tumor2, T3: Tumor3, T4: Tumor4, defined by the parameter values indicated in Table 1). Time evolution of A) proliferating, B) dead, C) terminally differentiated, D) stem, and E) LIMP (committed progenitor) cells.
(TIF)

Table S1 Miscellaneous model parameters (unrelated to tumor dynamics) and typical values where applicable.
(DOC)

Text S1 Calculation of reference values for the cell kill ratios of vincristine and actinomycin-D.
(DOC)

Text S2 Details regarding sensitivity analyses.
(DOC)
