# Supplementary figures and images for: Exploiting Clinical Trial Data Drastically Narrows the Window of Possible Solutions to the Problem of Clinical Adaptation of a Multiscale Cancer Model
Source: PLoS One. 2011 Mar 3;6(3):e17594. doi: 10.1371/journal.pone.0017594 (PMC3048172; doi:10.1371/journal.pone.0017594)

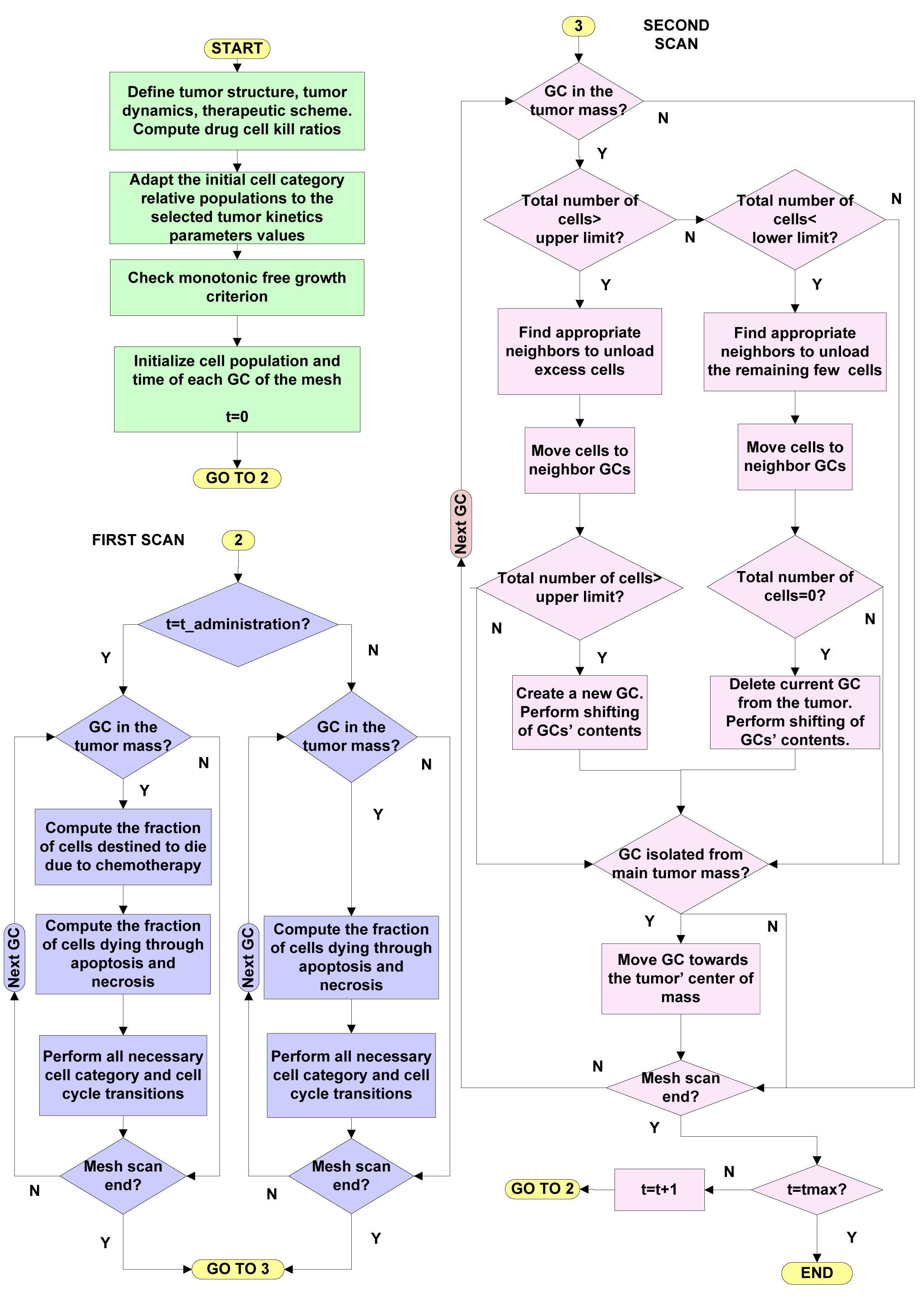

Supplement: Figure S1 — Simplified flowchart of the simulation procedure. Simplified flowchart of the simulation procedure. GC: Geometrical Cell. Y:Yes, N:No. (TIF) [file pone.0017594.s001.tif]

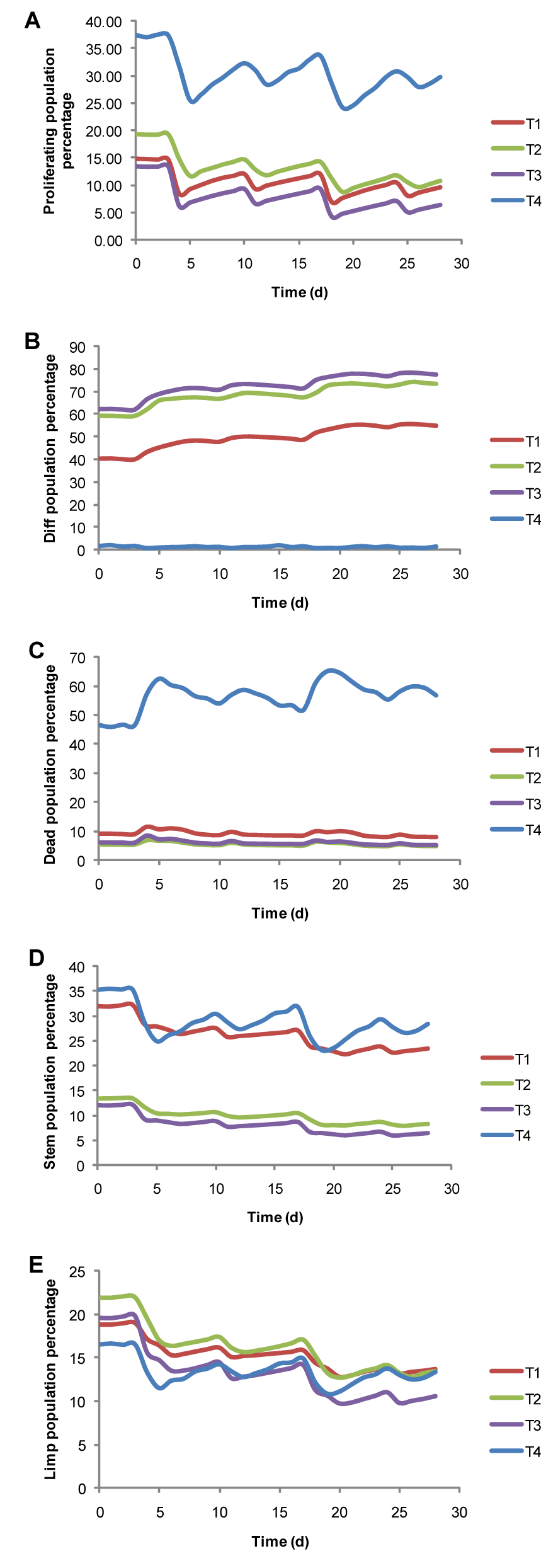

Supplement: Figure S2 — Time evolution of various tumor subpopulations. Alternative presentation of various tumor subpopulations for the four virtual tumor scenarios implemented (T1: Tumor1, T2: Tumor2, T3: Tumor3, T4: Tumor4, defined by the parameter values indicated in Table 1). Time evolution of A) proliferating, B) dead, C) terminally differentiated, D) stem, and E) LIMP (committed progenitor) cells. (TIF) [file pone.0017594.s002.tif]
