# Supplementary material for: Exploiting Clinical Trial Data Drastically Narrows the Window of Possible Solutions to the Problem of Clinical Adaptation of a Multiscale Cancer Model
Source: PLoS One. 2011 Mar 3;6(3):e17594. doi: 10.1371/journal.pone.0017594 (PMC3048172; doi:10.1371/journal.pone.0017594)
Supplement: Table S1 — Miscellaneous model parameters (unrelated to tumor dynamics) and typical values where applicable. (DOC) [file pone.0017594.s003.doc]

**Supporting Table S1:** Miscellaneous model parameters (unrelated to tumor dynamics) and typical values where applicable

| **Other model parameters** | | | |
| --- | --- | --- | --- |
| NBC | Number of biological cells typically contained within a GC of the mesh (assigned in relation to the GC’s volume) | 10^6^  (for GC volume=1mm^3^) | Based on typical tumor cell densities.  Error: Reference source not found |
| Margin percent | Acceptable temporary over-loading or under-loading of each geometrical cell as a fraction of unity | 0.1 | Based on exploratory runs |
| T_init_ (h) | Time interval between the pre-treatment imaging data acquisition and the first drugs’ administration | - | Clinical data or exploratory simulation data |
| Tpt_scan (h) | Time interval between the last drug administration and the post-treatment imaging data acquisition | - | Clinical data or exploratory simulation data |
| T_admin,i_ | Drug administration instants | - | Treatment protocol/clinical data |
