# Supplementary material for: Exploiting Clinical Trial Data Drastically Narrows the Window of Possible Solutions to the Problem of Clinical Adaptation of a Multiscale Cancer Model
Source: PLoS One. 2011 Mar 3;6(3):e17594. doi: 10.1371/journal.pone.0017594 (PMC3048172; doi:10.1371/journal.pone.0017594)
Supplement: Text S1 — Calculation of reference values for the cell kill ratios of vincristine and actinomycin-D. (DOC) [file pone.0017594.s004.doc]

**TEXT S1. Calculation of reference values for the cell kill ratios of vincristine and actinomycin-D.**

Published results of two independent studies have been used to acquire an approximate value for the Area Under Curve (AUC) of vincristine. According to the first study (S1), the AUC after a vincristine intravenous (i.v.) bolus injection of 1.5 mg/m^2^ s has been calculated equal to 6.7mg/L/min. The second study (S2) reached the conclusion that treatment with 16×10^-3^ μg/ml of vincristine for 6 hours produces an irreversible metaphase arrest and an AUC of 5.76 μg/ml/min =5.76 mg/L/min, which is very close to the clinical AUC that has been observed in (S1). The metaphase index calculated at 90 min after the removal of the drug (a time period during which it increases) was equal to 240 (cells stuck in metaphase per 1000 cells). The ratio of 240/1000=0.24 can be considered to reflect the cell kill fraction in the experiment, since mitosis cannot be completed, the cell cycle cannot proceed and death should follow. As the value of 5.76 μg/ml/min for the AUC in this experiment is slightly lower than the clinical AUC value of 6.7 mg/L/min, a cell kill fraction equal to 0.3 could be justified as an initial gross approximation. As a first approximation also the imperfect drug penetration into the tumor is assumed to have been taken into account in this value of 0.3 cell kill fraction. If a specific value of vincristine’s CKR is to be used in an exploratory simulation run or if a reference value of CKR is needed (see next sections) the above value of 0.3 is used.

Actinomycin- D is considered to be a less potent cytotoxic drug compared to vincristine, as indicated by lower AUC and higher IC50 values for various tumour and normal cells (S3)-(S4). Since recent literature data for dactinomycin pharmacokinetics proved to be rather scarce, a cell kill fraction equal to 0.2 (which is less than vincristine’s cell kill ratio) has been adopted as a starting point for a first approximation. Imperfect drug penetration into the tumour is assumed to have been taken into account when considering this cell kill fraction value. Again, if a specific value of actinomycin-D’s CKR is to be used in a simulation run or if a reference value of CKR is needed (see next sections) the above value of 0.2 is used.

Based on the above computation, the relationship CKR_Actinomycin-D_ =(2/3)*CKR_Vincristine_  is considered for parametric studies exploring ranges of CKR_total_ values.

Systematic use of SIOP/GPOH clinical trial data is expected to permit a quantitative refinement, if necessary, of the reference estimations of the cell kill ratios of vincristine and actinomysin-D.

[S1] Groninger E, Meeuwsen-de Boer T, Koopmans P, Uges D, Sluiter W, Veerman A, Kamps W, de Graaf S. Pharmacokinetics of Vincristine Monotherapy in Childhood Acute Lymphoblastic Leukemia. Pediatric Research 2002;52:113-118.

[S2] Dahl WN, Oftebro R, Pettersen EO, Brustad T. Inhibitory and cytotoxic effects of Oncovin (Vincristine Sulfate) on cells of human line NHIK 3025. Cancer Res. 1976;36:3101-3105.

[S3] Sawada K, Noda K, Nakajima H, Shimbara N, Furuichi Y, Sugimoto M. Differential cytotoxicity of anticancer agents in pre- and post-immortal lymphoblastoid cell lines. Biol Pharm Bull 2005;28:1202-1207.

[S4] Veal GJ, Cole M, Errington J, Parry A, Hale J, Pearson ADJ, et al. Pharmacokinetics of Dactinomycin in a pediatric patient population: a United Kingdom Children’s Cancer Study group study. Clin Cancer Res 2005;11(16): 5893-5899.
