# Supplementary material for: Exploiting Clinical Trial Data Drastically Narrows the Window of Possible Solutions to the Problem of Clinical Adaptation of a Multiscale Cancer Model
Source: PLoS One. 2011 Mar 3;6(3):e17594. doi: 10.1371/journal.pone.0017594 (PMC3048172; doi:10.1371/journal.pone.0017594)
Supplement: Text S2 — Details regarding sensitivity analyses. (DOC) [file pone.0017594.s005.doc]

**TEXT S2. Details regarding sensitivity analyses**

The sensitivity analysis approach adopted for the sorting of model’s parameters in terms of their effect on the simulation outcome was the consideration of a ±5% variation (variation factor h=0.05) around the reference value of each studied model parameter and subsequent inspection of the variation in the output.

The Sorting Criterion, SC, was defined by:

SC= (|dV_i_|/V_ref_)/(|dp_i_/p_iref_|), i=1,…,12. (Equation S1)

where p_1ref_,p_2ref_, …,p_iref_,…,p_12ref_ are the reference values of the model parameters, V_ref_ is the final tumor volume obtained with the reference value p_iref_ of the parameter p_i_, dV_i_= V(p_1ref_,p_2ref_, …,p_iref_+h*p_iref_,…,p_12ref_)-V(p_1ref_,p_2ref_, …,p_iref_-h*p_iref_,…,p_12ref_) is the difference in the final tumor volume between the two runs (one with p_iref_+h*p_iref_ and the other with p_iref_-h*p_iref_), and dp_i_ is the difference in the two values of the parameter p_i_. Since:

dp_i_ = p_iref_ + h* p_iref_ –( p_iref_ - h* p_iref_)=2*h*p_iref_ (Equation S2)

the sorting criterion finally becomes:

SC = (|dV_i_|/V_ref_)/2*h (Equation S3)

In all simulations, the special case of macroscopically homogeneous tumors has been considered for simplification purposes, implying that various model parameter values refer actually to their spatial average throughout the tumor. In addition, the use of constant values of the tumor dynamics parameters throughout the simulation is considered as a plausible approximation, bearing in mind the narrow time window of the chemotherapy treatment duration.

For tumor regrowth after therapy studies, an exponential free growth pattern has been considered, which in fact approximates a segment of the Gompertzian curve. Under this assumption, for a short time interval the evolution of the tumor’s volume over time can be described by the equation:

V(t)=V_0_e^kt^ (Equation S3)

where V_0_ is the volume at t=0 and k is the growth rate constant. The growth rate constant of the tumors has been computed by least squares fit regression analysis.
